# The causal association between bone mineral density and risk of osteoarthritis: A Mendelian randomization study

**DOI:** 10.3389/fendo.2022.1021083

**Published:** 2023-01-11

**Authors:** Liying Jiang, Ying Jiang, Anqi Wang, Cui Wu, Yi Shen

**Affiliations:** ^1^ Department of Prevention Medicine, College of Public health, Shanghai University of Medicine & Health Sciences, Shanghai, China; ^2^ Jiading Central Hospital, Shanghai University of Medicine & Health Sciences, Shanghai, China; ^3^ Department of Health, Center for Disease Control and Prevention in Suqian, Suqian, Jiangsu Province, China; ^4^ Department of Nursing, Shanghai University of Traditional Chinese Medicine, Shanghai, China; ^5^ Department of Non-communicable Disease, Baoshan District Center for Disease Control and Prevention in Shanghai, Shanghai, China; ^6^ Department of Epidemiology, School of Public Health, Nantong University, Nantong, Jiangsu Province, China

**Keywords:** osteoarthritis, bone mineral density, single nucleotide polymorphisms, Mendelian randomization, genome-wide association studies

## Abstract

**Objectives:**

The causal direction and magnitude of the association between total body bone mineral density (TB-BMD) and osteoarthritis (OA) risk is uncertain owing to the susceptibility of observational studies to confounding and reverse causation. The study aimed to explore the relationships between TB-BMD concentration and OA using Mendelian randomization (MR).

**Methods:**

In this study, we used two-sample MR to obtain unconfounded estimates of the effect of TB-BMD on hip and knee OA. Single nucleotide polymorphisms (SNPs) strongly associated with TB-BMD in a large genome-wide association study (GWAS) were identified and selected as instrumental variables (IVs). In addition to the main analysis using inverse-variance weighted (IVW) method, we applied 2 additional methods to control for pleiotropy(MR-Egger regression, weighted median estimator) and compared the respective MR estimates.

**Results:**

MR analyses suggested that genetically predicted higher TB-BMD is associated with risks of hip OA (For IVW: OR=1.199, 95%CI: 1.02-1.42, *P*=0.032; for WM: OR=1.257, 95%CI: 1.09-1.45, *P*=0.002). There was no evidence that the observed causal effect between TB-BMD and the risk of hip OA was affected by genetic pleiotropy(*P*=0.618). Additionally, our study didn’t support causal effects of a genetically increased TB-BMD risk on knee OA risk(OR=1.121, 95%CI: 0.99-1.28, *P*=0.084 using IVW; OR=1.132, 95%CI: 0.99-1.29, *P*=0.068 using WM; OR=1.274, 95%CI: 0.88-1.85, *P*=0.217 using MR-Egger).

**Conclusions:**

Our findings support a causal effect that a genetic predisposition to systematically higher TB-BMD was associated with the risk of OA. And, TB-BMD likely exerts an effect on the risk of hip OA not knee OA.

## Introduction

Osteoarthritis (OA) is the most prevalent musculoskeletal disease and the most common form of arthritis (1, 2). The hallmarks of OA are the degeneration of articular cartilage, remodeling of the underlying bone and synovial (3). As the leading cause of disability worldwide, it affects 40% of individuals over 70 years old and carries a substantial public health and health economic burden (4, 5). The health economic burden of OA is continually rising, commensurate with longevity and obesity rates, and current disease-modifying treatment is minimally effective (6). As a highly heterogeneous disease, the pathological features of OA include pain, inflammation, cartilage degradation and bone spurs. The heritability of the disease is over 50%, and previous genetic studies have identified 21 loci in total across hip, knee, and hand sites with limited overlap (5).

Bone mineral density (BMD) is considered to be one of the integral influences in the pathogenesis of OA. The association between BMD and OA was first reported by Foss in 1972 when they observed that higher BMD in femoral heads resected during OA-related hip replacement surgery (7). A cross-sectional study suggested that higher BMD was associated with an increased risk of incident OA defined by osteophyte or Kellgren-Lawrence(KL) grade, suggesting that increased BMD is a risk factor for OA development (8). Women with radiographic hip OA had a 9%-10% higher BMD of the femoral neck compared to those without OA (9). Previous studies indicated that male hand OA patients have reduced radial trabecular density and thickness in the distal radius (10).

Genome-wide association studies (GWAS) have made an important contribution to the identification of genetic variants associated with numerous potential risk factors for health-related outcomes. The GWAS results have facilitated the use of Mendelian randomization(MR) to evaluate causal relationships making use of summary-level data from GWAS between modifiable exposures and outcomes (11). MR uses genetic variants as instrumental variables(IVs) to detect whether the exposure phenotype has a causal effect on the outcome phenotype (12). This approach can overcome common pitfalls of traditional research, such as confounding factors and reverse causality (13). MR method has been applied to estimate the causal associations between parathyroid Hormone (14), insulin-like growth factor-1(IGF-1) (15), and smoking behavior (16), and OA.

Compelling evidence has suggested that a causal effect of high femoral neck BMD on the risk of knee OA and hip OA is predominantly reflecting cortical bone mass (17). However, these studies are susceptible to confounding and reverse causation, and thus it remains unclear whether these associations are accurate. Inferring causality from such studies is challenging because it is difficult to exclude reverse causality, confounding, or measurement error. The relationship between BMD and OA has always been a controversial issue, especially for different sites. The identification of risk factors for primary prevention is therefore of major interest as a method to reduce the incidence and consequences of the disease. Understanding the role of BMD in the pathogenesis of OA may have important therapeutic significance, and this would be of clinical relevance for OA prevention in high-risk individuals since lower total body BMD(TB-BMD) is common and safely correctable in elderly adults.

To the best of our knowledge, the role of TB-BMD in OA risk has not been well established through observational studies. Herein, we used genetic variations strongly associated with TB-BMD traits as unconfounded instruments to investigate the potential causal effect of TB-BMD on the risk of integrating knee OA and hip OA in individuals of similar European origin. In our study, we applied two-sample MR, an approach that combines summary statistics on the genetic variant to exposure and genetic variant to outcome associations from different samples, to provide estimates on the strength of the association between exposure and outcome.

## Materials and methods

### Study overview

This study determined whether TB-BMD was causally related to OA using MR analysis. First, the genetic variants utilized as instrumental variables (IVs) should not be associated with confounders. Second, genetic variants should not be associated with confounders. Third, the genetic variants should affect the risk of the outcome through the risk factor, not *via* other pathways. The MR analysis followed strictly the STROBE-MR Statement (18).

**Figure 1:**
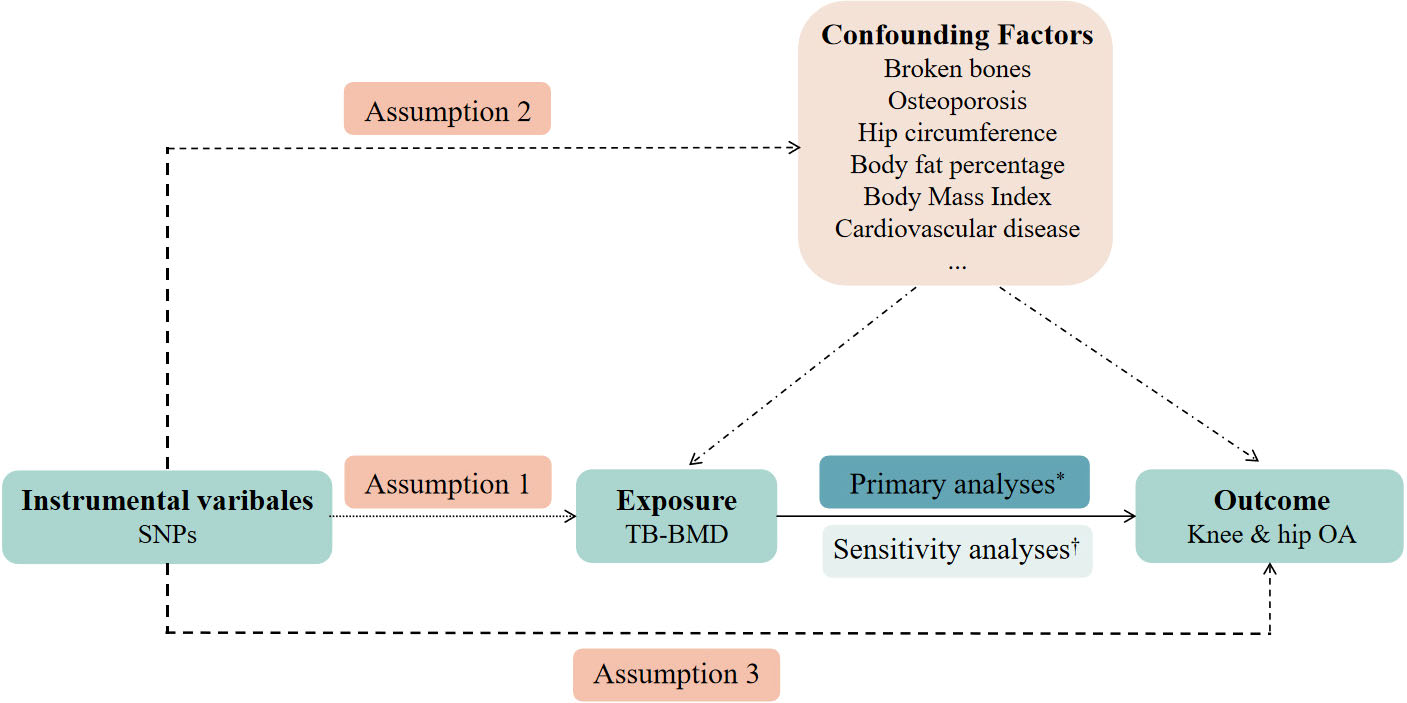
An overview of the study design. SNP, single-nucleotide polymorphism; TB-BMD, total body bone mineral density; OA, osteoarthritis. Assumption 1: the instrumental variables (IVs) must be robustly associated with the exposure. Assumption 2: the IVs must not be associated with any confounding factors of the exposure-outcome relationship. Assumption 3: the IVs must only affect the outcome through the exposure. *Primary analyses: inverse-variance weighted (IVW) method, weighted median estimator, and MR-Egger regression. †Sensitivity analyses: cochran’s Q test and leave-one-out analyses.

As this study is based on existing publications and public databases, no additional ethical approval or consent to participate is required. Further details on the design strategy are shown in **Figure 1**.

### Selection of genetic variants

Publicly accessible data for genetic variants associated with TB-BMD were retrieved obtained from the GEnetic Factors for OSteoporosis(GEFOS) consortium (http://www.gefos.org/). Summary data from a previous meta-analysis of 30 GWASs of TB-BMD including 66,628 individuals (56,284 individuals of European ancestry) was performed to investigate genetic determinants of TB-BMD variation (19). The SNPs of TB-BMD were already corrected covariates (age, weight, height and so on) *via* linear regression models (19).

Screening criteria for TB-BMD-associated SNPs: (i)SNPs have significance in genome-wide studies(*P*<5×10^−8^). (ii)All IVs were independent with linkage disequilibrium(LD)(*r* ^2^<0.001), which validated the independence of selected genetic variants (20).

### OA GWAS summary statistics

We searched the NHGRI-EBI GWAS catalog (www.ebi.ac.uk/gwas), which is a manually curated collection of complete GWAS summary datasets. Summary statistics for the effect of TB-BMD-associated SNPs on OA were derived from a genome-wide meta-analysis that included up to 455,221 European individuals (77,052 cases and 378,169 controls), complying with genotype data across 17.5 million variants from the UK Biobank and Arthritis Research UK OA Genetics(arcOGEN) resources (21).

### Statistical analysis

The MR approach was used to identify the potential causal link of TB-BMD with knee OA and hip OA. All statistical data analyses were carried out using the package “TwoSampleMR” of the R program (version 4.1.0). The two-sample MR method was estimated for the genetically causal associations were obtained by applying the inverse-variance weighted (IVW) analysis, weighted median(WM) estimator, and MR-Egger regression (22–25). The IVW method examines the causal link by performing a meta-analysis of each Wald ratio for the included SNPs (23). Significantly, the IVW analysis is predicated on the assumption that all of the contained SNPs are genuine variables (23). Unlike the IVW method, the MR-Egger regression can work even if all of the SNPs are invalid. However, MR-Egger may be inaccurate, especially when the correlation coefficient between SNPs and the exposure is similar or the number of genetic instruments is small (22). The WM estimator produced the median when the effect estimations of each single SNP were sorted in order of weight values (24). The estimation of the causal relationship between TB-BMD and OA was expressed as odds ratio(OR) and 95% confidence interval(CI). In addition, *P*<0.05 was the threshold for a significant difference.

MR analysis assumes that the selected IVs should act on the outcome only through the exposure variable. To eliminate the impact of known confounding factors on causality estimates, potential pleiotropic effects of selected SNPs on BMD were verified *via* the Ensembl project (http://www.ensembl.org) and the PhenoScanner (http://www.phenoscanner.medschl.cam.ac.uk). We systematically checked each SNP on the both websites and comprehensively aggregated the results. Finally, we excluded one SNP(rs2043230) at-a-time and performed analysis on the remaining SNPs to identify outlying IVs.

### Heterogeneity and sensitivity test

In analyses of two-sample MR, pleiotropy represents those genetic variants or SNPs with multiple effects. That is, pleiotropic genetic variants or SNPs may have an effect on the outcome, not the exposure, which could cause a bias in the MR estimate and potential confounding effects; thus, investigating pleiotropy is essential. In the study, we removed SNPs one by one using the “leave-one-out” analysis and calculated the combined effect of the remaining SNPs separately to determine the magnitude of the effect of each SNP on the results. If the results of the “leave-one-out” analysis are inconsistent with the results of the causal effects analysis, it indicates that there may be effect on the estimated causal effects (22). Also, heterogeneity test is essential. We used Cochran’s Q statistics to explore heterogeneity among these SNPs (26). In cases where Cochran’s Q test indicated there was pleiotropy, we adopted the results of a random effects model instead of a fixed effects IVW model. For the sensitivity test, we performed a subgroup analysis utilizing only IV SNPs with genome-wide significance(*P*<5×10^−8^).

## Results

The detail of studies and datasets was presented in **Table 1**. The participants were all of European(100%), overcoming the issue of ethnic differences.

**Table 1:** Details of Studies and Datasets Used in the Study.

Exposure/Outcome	Sample Size	Web Source	First Author	Consortium	Year	Population
TB-BMD	56,284	http://www.gefos.org/	Medina C	GEFOS	2018	European
Knee OA	403,124	https://gwas.mrcieu.ac.uk/	Tachmazidou I	–	2019	European
Hip OA	393,873	https://gwas.mrcieu.ac.uk/	Tachmazidou I	–	2019	European

TB-BMD, total body bone mineral density; OA, osteoarthritis; GEFOS, the GEnetic Factors for OSteoporosis.

### Characteristics of instrumental variables

The original data of IVs can be obtained from the link (https://www.ncbi.nlm.nih.gov/pmc/articles/PMC5777980/). Genetic variants were screened according the criteria of screening(*P*<5×10^−8^, *r* ^2^<0.001, kb=10000). Eventually, 29 SNPs were employed as IVs for TB-BMD in the present study (**Table 2**). The associations between IVs for knee OA and hip OA are summarized in **Supplementary Tables 1, 2**, including the chromosome location, genes, effect allele(EA), other allele and effect allele frequency(EAF). In addition, estimations of the association between each SNP with TB-BMD and OA, including beta value, standard error(SE) and *P* value, were also presented.

**Table 2:** Characteristics of SNPs for TB-BMD.

SNPs	Chr	Effect allele	Other allele	EAF	Beta	SE	*P*-value
rs10249736	7:120737177	A	G	0.45	-0.0370	0.0062	2.25E-09
rs10777212	12:90334829	T	G	0.35	0.0454	0.0066	5.00E-12
rs10838622	11:46856536	T	C	0.36	0.0491	0.0067	3.04E-13
rs117557198	12:49655948	A	G	0.93	-0.0887	0.0128	3.84E-12
rs11910328	21:40350744	A	G	0.84	-0.0489	0.0085	8.51E-09
rs12293302	11:15776444	A	T	0.03	0.1441	0.0209	5.05E-12
rs12612325	2:119632252	A	G	0.20	-0.0600	0.0086	2.95E-12
rs1286079	14:91445162	T	C	0.19	0.0552	0.0080	5.42E-12
rs1385162	11:15689391	A	G	0.21	0.0466	0.0077	1.24E-09
rs144279715	2:119548256	A	G	0.98	-0.2178	0.0309	1.89E-12
rs1452102	21:28773868	T	G	0.58	-0.0343	0.0062	3.29E-08
rs2043230	2:85483350	A	T	0.44	0.0339	0.0062	4.77E-08
rs2289410	2:42284110	A	T	0.87	0.0528	0.0096	4.05E-08
rs344024	3:156474152	A	G	0.77	0.0504	0.0072	3.11E-12
rs3757493	7:96656572	T	G	0.42	-0.0359	0.0063	1.28E-08
rs6716216	2:202803881	A	G	0.88	-0.0658	0.0095	4.71E-12
rs6965122	7:96133319	A	G	0.68	0.0766	0.0066	4.64E-31
rs71390846	16:86714715	C	G	0.19	-0.0498	0.0081	6.95E-10
rs73305797	7:30997087	A	T	0.75	0.0424	0.0072	3.71E-09
rs7364724	1:110480220	A	G	0.40	-0.0377	0.0063	1.84E-09
rs73719811	7:121200844	T	C	0.93	-0.0944	0.0130	3.42E-13
rs746627	17:63850776	T	C	0.32	0.0414	0.0069	1.56E-09
rs7586085	2:166577489	A	G	0.52	0.0511	0.0062	1.72E-16
rs7740042	6:151971720	A	T	0.20	-0.0552	0.0077	7.86E-13
rs7741085	6:44636919	T	C	0.59	0.0465	0.0063	1.19E-13
rs78667121	13:43200103	A	G	0.03	0.1557	0.0192	5.96E-16
rs8047501	16:392318	A	G	0.49	0.0559	0.0065	6.83E-18
rs884205	18:60054857	A	C	0.25	-0.0530	0.0073	3.96E-13
rs9976876	21:36970350	T	G	0.46	-0.0381	0.0063	1.35E-09

TB-BMD, total body bone mineral density; SNP, single nucleotide polymorphism; EAF, effect allele frequency; SE, standard error.

### Causal association with knee OA and hip OA

One palindromic SNPs (rs2043230) with intermediate allele frequencies was removed. Thus, 28 independent SNPs associated with TB-BMD in European ancestry were chosen to perform the MR analysis for the causal link between TB-BMD and knee OA and hip OA. None of the individual 28 SNPs was associated with knee OA in the study(OR: 1.121, 95%CI: 0.99-1.28, *P*=0.084 using IVW; OR: 1.132, 95%CI: 0.99-1.29, *P*=0.068 using WM; OR: 1.274, 95%CI: 0.88-1.85, *P*=0.217 using MR-Egger)(shown in **Table 3**).

**Table 3:** MR estimates of associations between TB-BMD with knee and hip OA using various analysis methods.

Exposure	MR method	Number of SNPs	OR	95% CI	Association *P*-value	Cochran’s Q Statistic	Heterogeneity *P*-value
Knee OA							
TB-BMD	IVW	28	1.121	0.99-1.28	0.084	76.61	0.484
	WM	28	1.132	0.99-1.29	0.068		
	MR-Egger	28	1.274	0.88-1.85	0.217		
Hip OA							
TB-BMD	IVW	28	1.199	1.02-1.42	0.032^*^	79.22	0.618
	WM	28	1.257	1.09-1.45	0.002^*^		
	MR-Egger	28	1.347	0.83-2.18	0.235		

^*^Statistically significant P-value.

MR, Mendelian randomization; TB-BMD, total body bone mineral density; OA, osteoarthritis; SNP, single nucleotide polymorphism; IVW, inverse variance weighted; WM, weighted median.

**Figure 2:**
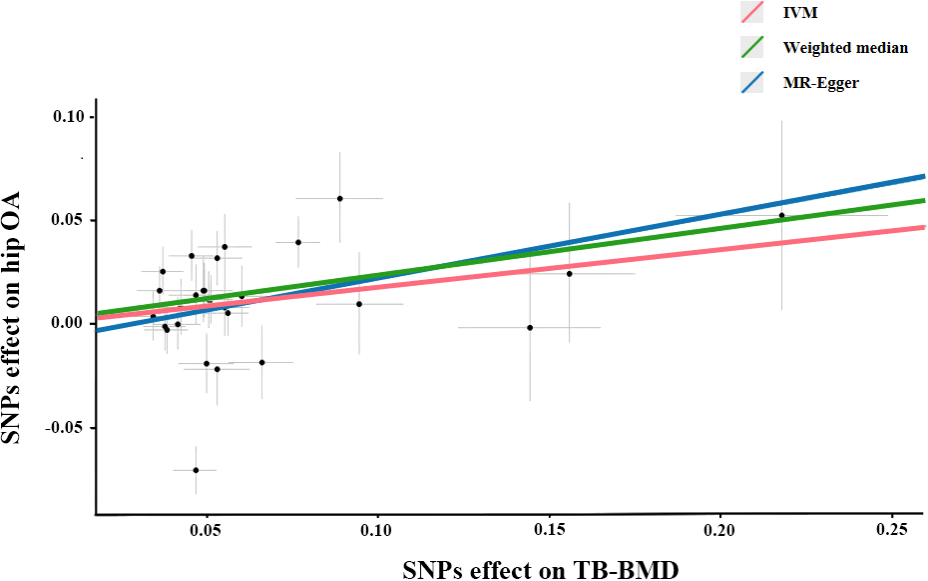
Scatter plots of the Mendelian randomization analyses for the association of TB-BMD with the risk of hip OA. The red line represents the inverse variance weighted estimate, the green line represents the Weighted median estimate, the blue line represents the MR-Egger estimate.

The association between genetically predicted TB-BMD and hip OA were detected using the IVW method (OR=1.199), although with a wider CI(95%CI: 1.02-1.42, *P*=0.032). The association between genetically predicted TB-BMD and hip OA was consistent with IVW method using WM method (OR=1.257, 95%CI=1.09-1.45, *P*=0.002) (**Table 3**; **Figure 2**). There was no causal association between TB-BMD and hip OA using MR-Egger analysis(OR: 1.347, 95% CI: 0.83-2.18, *P*=0.235), and this was inconsistent with WM and IVM methods.

### Sensitivity analysis

**Figure 3:**
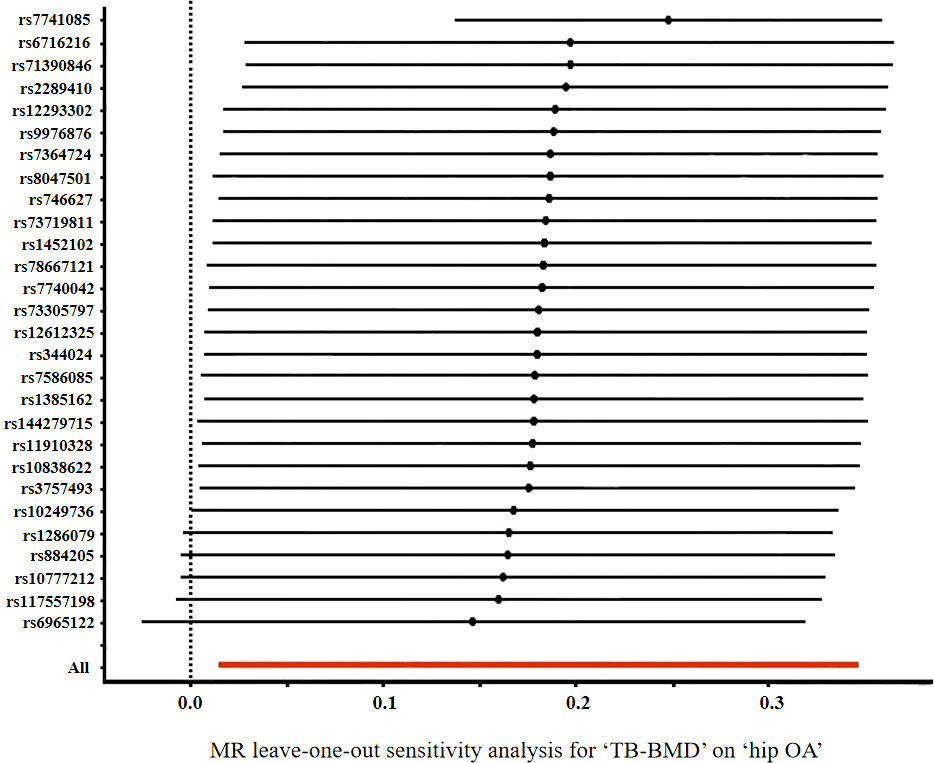
Forrest plot of the causal effects of TB-BMD-associated single nucleotide polymorphisms (SNPs) on hip OA.

According to Cochran’s Q test, there was no evidence of heterogeneity between the MR estimates based on individual variants using IVW method (For knee OA, Q value=76.61, *P*=0.484; for hip OA, Q value=79.22, *P*=0.618) (**Table 3**). The “leave one out” analysis revealed that no single SNP play a decisive role in the causal inference (**Figure 3**).

## Discussion

The causal direction and magnitude of the association between TB-BMD and risk of OA is uncertain, and observational studies make up the majority of previous research. Observational studies are susceptible to demonstrate confounding, reverse causation bias, and measurement error, all of which limit their ability to provide causal estimates of the effect of exposures on outcomes, thereby reducing their ability to inform prevention and treatment strategies against the disease. Unlike observational studies, MR uses exceptional genetic variants that are assumed to satisfy the IVs hypothesis to investigate the question of causality in epidemiological studies, which minimizes the possibility of inherent bias (27). Compared with randomized controlled trials, MR analysis is cost-effective and feasible (28).

In this study, we explored the relationship between TB-BMD and OA risk *via* a two-sample MR analysis based on 28 SNPs significantly related to TB-BMD as genetic variants. Despite the fact that the MR estimates from IVW, MR-Egger, and WM analysis were inconsistent, the IVW and WM estimators support a causal inverse relationship between TB-BMD and hip OA. The MR analysis suggests that a causal relationship between TB-BMD and hip OA, as the weighted average retains greater precision in the estimates compared to the MR-Egger analysis. However, there is no evidence of a causal association between TB-BMD and knee OA based on these three analyses. MR provide insights into the nature of the genetic aetiology we observed. Large-scale whole genome sequencing studies of well phenotyped individuals across diverse populations will capture the full allele frequency and variation type spectrum, and prompt us to explore the causes of this debilitating disease. This MR approach offers an alternative analytical technique being able to reduce conventional patterns of confounding and reverse causation and re-estimate observations in a framework allowing causal inference. These findings present important novel evidence on the etiology of OA.

OA was previously thought to be a cartilage disease, whereas it is now considered as a disease of the entire joint with other articular components playing a key role in the pathogenesis of OA (29). The central feature of OA is joint cartilage degradation and loss, which is manifested radiographically as a narrowing of the joint space. Because of cartilage loss and metabolic disturbances, the biomechanics of the joint change during the process of OA development result in altered subchondral bone elasticity and stiffness (30). Possible mechanisms whereby changed BMD in the subchondral bone(the region of bone immediately beneath the cartilage that includes the cortical plate and the subchondral cancellous bone) and altered gene expression might be potential players in cartilage degeneration pathogenesis (31). The potential mechanisms underlying the association need to be more thoroughly evaluated.

The reliability of the findings from a MR study depends on 3 key assumptions, which could be violated by population stratification, canalization, pleiotropy and linkage disequilibrium. Since not all genetic variants used as proxies for the exposure of interest may fulfill the “no pleiotropy” assumption, several approaches were undertaken to assess and adjust for potential confounding or pleiotropic effects. Sensitivity analyses using Cochran’s Q statistics and the “leave-one-out” method to explore and adjust for pleiotropy were also conducted. Pleiotropy is defined as the phenomenon in which a single locus affects two or more distinct phenotypic traits, resulting in compromises between trait adaptations because a mutation that benefits one trait may harm another (32, 33). The credibility of MR study results is likely to be affected by whether pleiotropy leads to bias. The results were consistent across these analyses, indicating that confounding is unlikely to explain the observed associations. Linkage disequilibrium with directly causal variants (violating the third assumption) was likely avoided owing to the use of multiple SNPs, most of which were positively associated with hip OA.

Recently, the temporal relationship between BMD and OA has been confirmed. In Johnston County, North Carolina (NC), higher BMD was associated with a lower risk of hip OA among adults aged 45 years old. Intermediate, but not high, BMD levels were linked to an increased risk of knee and hip OA (34). A case-control study provides strong evidence that high baseline femoral neck BMD is significantly related to the incidence of knee and hip OA, but not the incidence of hand OA. Whereas high baseline BMD is not associated with the progression of knee OA (35). Both men and women have presented lower fracture risk when their BMD is higher, particularly in the hip site (36). In middle-aged and older adults, lower fracture risk may be offset by an increased risk of incident knee OA (37). In postmenopausal women, there has been a similar link between higher BMD and incident radiographic hip OA (38). These studies are consistent with the results of the causal analysis.

The strengths of this MR analysis include the availability of larger sample size and the use of multiple uncorrelated SNPs associated with BMD based on published GWAS meta-analysis data, which increase the precision of the estimates. Moreover, a two-sample MR obtains IV-exposure and IV-outcome associations from two different sets of participants. Two-sample MR that uses the effect of IVs on the exposure and the outcome phenotype from two independent studies can greatly increase the power of detecting causality compared with a single sample study (39). Most importantly, population stratification arises when different subgroups of the population vary substantially. This bias can be mitigated by limiting the analysis to ethnically homogenous groups. The participants contributing to this study were composed of European descent populations, and the SNPs have been consistently associated with BMD in populations of different ancestries.

This study has several limitations that need to be considered. First, the overall estimate of the genetic association was based on a publicly available summary meta-analysis, and the study lacked complete information on sex and age. Therefore, subgroup analyses could not be conducted to explore the association separated by study-specific characteristics(e.g. age, gender), and a potential non-linear association between serum BMD levels and OA could not be further evaluated. A potential limitation is that datasets with a larger number of OA cases and non-cases were unavailable in the study. Genetic variants generally have small effects on the exposure and possibly explain only a small proportion of the variance, and large sample sizes are necessary to obtain statistically significant results. Other study limitations include an inability to exclude the existence of smaller associations and a lack of evidence from non-European populations.

In conclusion, our findings provide some support for a causal effect, whereby a systematically higher genetic susceptibility to TB-BMD is associated with an increased risk of OA. The use of multiple SNPs in MR analysis has also established that increased BMD is associated with the risk of hip OA, but not with an increased knee OA risk. Whether the risk of hip OA associated with lifelong genetic exposure to increased BMD can be translated into a risk associated with short-term to medium-term body load is unknown. Given that TB-BMD status is a modifiable trait, these results may have clinical and public health implications that need confirmation by further larger MR studies and clinical trials.

## Data availability statement

The datasets presented in this study can be found in online repositories. The names of the repository/repositories and accession number(s) can be found in the article/**Supplementary Material**.

## Author contributions

LJ drafted the protocol and wrote the final paper. YJ contributed to data analysis and interpretation of results. YS made critical revisions. AW and CW participated in the data collection. All authors contributed to the article and approved the submitted version.
