# Supplementary material for: The causal association between bone mineral density and risk of osteoarthritis: A Mendelian randomization study
Source: Front Endocrinol (Lausanne). 2023 Jan 11;13:1021083. doi: 10.3389/fendo.2022.1021083 (PMC9874138; doi:10.3389/fendo.2022.1021083)
Supplement: Supplementary file 1 [file DataSheet_1.docx]

| Supplementary Table 1. Characteristics of SNPs for knee OA. | | | | | | | |
| --- | --- | --- | --- | --- | --- | --- | --- |
| **SNPs** | **Chr** | **Effect allele** | **Other allele** | **EAF** | **Beta** | **SE** | ***P*-value** |
| rs10249736 | 7:120737177 | G | A | 0.45 | 0.0009 | 0.0093 | 9.20E-01 |
| rs10777212 | 12:90334829 | T | G | 0.35 | 0.0203 | 0.0098 | 3.86E-02 |
| rs10838622 | 11:46856536 | C | T | 0.36 | -0.0111 | 0.0103 | 2.81E-01 |
| rs117557198 | 12:49655948 | G | A | 0.93 | -0.0176 | 0.0174 | 3.14E-01 |
| rs11910328 | 21:40350744 | A | G | 0.84 | -0.0249 | 0.0128 | 5.16E-02 |
| rs12293302 | 11:15776444 | A | T | 0.03 | -0.0230 | 0.0293 | 4.33E-01 |
| rs12612325 | 2:119632252 | A | G | 0.20 | -0.0404 | 0.0120 | 7.80E-04 |
| rs1286079 | 14:91445162 | T | C | 0.19 | 0.0129 | 0.0123 | 2.95E-01 |
| rs1385162 | 11:15689391 | G | A | 0.21 | 0.0222 | 0.0116 | 5.51E-02 |
| rs144279715 | 2:119548256 | G | A | 0.98 | 0.0813 | 0.0364 | 2.55E-02 |
| rs1452102 | 21:28773868 | G | T | 0.58 | -0.0008 | 0.0094 | 9.34E-01 |
| rs2043230 | 2:85483350 | T | A | 0.44 | 0.0150 | 0.0094 | 1.11E-01 |
| rs2289410 | 2:42284110 | T | A | 0.87 | -0.0064 | 0.0140 | 6.47E-01 |
| rs344024 | 3:156474152 | G | A | 0.77 | -0.0193 | 0.0109 | 7.61E-02 |
| rs3757493 | 7:96656572 | T | G | 0.42 | -0.0156 | 0.0094 | 9.53E-02 |
| rs6716216 | 2:202803881 | G | A | 0.88 | 0.0089 | 0.0142 | 5.29E-01 |
| rs6965122 | 7:96133319 | G | A | 0.68 | 0.0014 | 0.0099 | 8.86E-01 |
| rs71390846 | 16:86714715 | C | G | 0.19 | 0.0295 | 0.0119 | 1.32E-02 |
| rs73305797 | 7:30997087 | T | A | 0.75 | 0.0242 | 0.0106 | 2.28E-02 |
| rs7364724 | 1:110480220 | G | A | 0.40 | -0.0089 | 0.0094 | 3.47E-01 |
| rs73719811 | 7:121200844 | C | T | 0.93 | -0.0105 | 0.0197 | 5.93E-01 |
| rs746627 | 17:63850776 | T | C | 0.32 | 0.0107 | 0.0101 | 2.89E-01 |
| rs7586085 | 2:166577489 | G | A | 0.52 | -0.0200 | 0.0093 | 3.06E-02 |
| rs7740042 | 6:151971720 | A | T | 0.20 | -0.0242 | 0.0113 | 3.32E-02 |
| rs7741085 | 6:44636919 | T | C | 0.59 | -0.0233 | 0.0094 | 1.30E-02 |
| rs78667121 | 13:43200103 | A | G | 0.03 | 0.0566 | 0.0267 | 3.40E-02 |
| rs8047501 | 16:392318 | G | A | 0.49 | -0.0038 | 0.0094 | 6.82E-01 |
| rs884205 | 18:60054857 | C | A | 0.25 | 0.0365 | 0.0107 | 6.69E-04 |
| rs9976876 | 21:36970350 | T | G | 0.46 | 0.0106 | 0.0093 | 2.55E-01 |

**Abbreviations:** OA, osteoarthritis; SNP, single nucleotide polymorphism; EAF, effect allele frequency; SE, standard error.
