# Supplementary material for: The causal association between bone mineral density and risk of osteoarthritis: A Mendelian randomization study
Source: Front Endocrinol (Lausanne). 2023 Jan 11;13:1021083. doi: 10.3389/fendo.2022.1021083 (PMC9874138; doi:10.3389/fendo.2022.1021083)
Supplement: Supplementary file 2 [file DataSheet_2.docx]

| Supplementary Table 2. Characteristics of SNPs for hip OA. | | | | | | | |
| --- | --- | --- | --- | --- | --- | --- | --- |
| **SNPs** | **Chr** | **Effect allele** | **Other allele** | **EAF** | **Beta** | **SE** | ***P*-value** |
| rs10249736 | 7:120737177 | G | A | 0.45 | 0.0258 | 0.0117 | 2.70E-02 |
| rs10777212 | 12:90334829 | T | G | 0.35 | 0.0333 | 0.0123 | 6.96E-03 |
| rs10838622 | 11:46856536 | C | T | 0.36 | -0.0167 | 0.0133 | 2.09E-01 |
| rs117557198 | 12:49655948 | G | A | 0.93 | 0.0609 | 0.0219 | 5.34E-03 |
| rs11910328 | 21:40350744 | A | G | 0.84 | -0.0165 | 0.016 | 3.03E-01 |
| rs12293302 | 11:15776444 | A | T | 0.03 | -0.0013 | 0.0362 | 9.72E-01 |
| rs12612325 | 2:119632252 | A | G | 0.20 | -0.0136 | 0.0151 | 3.66E-01 |
| rs1286079 | 14:91445162 | T | C | 0.19 | 0.0378 | 0.0154 | 1.40E-02 |
| rs1385162 | 11:15689391 | G | A | 0.21 | -0.0142 | 0.0145 | 3.27E-01 |
| rs144279715 | 2:119548256 | G | A | 0.98 | 0.0525 | 0.0456 | 2.50E-01 |
| rs1452102 | 21:28773868 | G | T | 0.58 | 0.0041 | 0.0118 | 7.30E-01 |
| rs2043230 | 2:85483350 | T | A | 0.44 | -0.0298 | 0.0118 | 1.15E-02 |
| rs2289410 | 2:42284110 | T | A | 0.87 | 0.0216 | 0.0176 | 2.19E-01 |
| rs344024 | 3:156474152 | G | A | 0.77 | -0.0119 | 0.0136 | 3.82E-01 |
| rs3757493 | 7:96656572 | T | G | 0.42 | -0.0162 | 0.0118 | 1.69E-01 |
| rs6716216 | 2:202803881 | G | A | 0.88 | -0.0182 | 0.0178 | 3.06E-01 |
| rs6965122 | 7:96133319 | G | A | 0.68 | -0.0396 | 0.0124 | 1.43E-03 |
| rs71390846 | 16:86714715 | C | G | 0.19 | 0.0187 | 0.0149 | 2.10E-01 |
| rs73305797 | 7:30997087 | T | A | 0.75 | -0.0084 | 0.0134 | 5.31E-01 |
| rs7364724 | 1:110480220 | G | A | 0.40 | -7.00E-04 | 0.0118 | 9.55E-01 |
| rs73719811 | 7:121200844 | C | T | 0.93 | 0.0099 | 0.0247 | 6.87E-01 |
| rs746627 | 17:63850776 | T | C | 0.32 | 4.00E-04 | 0.0127 | 9.77E-01 |
| rs7586085 | 2:166577489 | G | A | 0.52 | -0.012 | 0.0116 | 3.02E-01 |
| rs7740042 | 6:151971720 | A | T | 0.20 | -0.0084 | 0.0142 | 5.57E-01 |
| rs7741085 | 6:44636919 | T | C | 0.59 | -0.0704 | 0.0118 | 2.19E-09 |
| rs78667121 | 13:43200103 | A | G | 0.03 | 0.0247 | 0.0337 | 4.62E-01 |
| rs8047501 | 16:392318 | G | A | 0.49 | -0.0058 | 0.0117 | 6.24E-01 |
| rs884205 | 18:60054857 | C | A | 0.25 | 0.0319 | 0.0135 | 1.79E-02 |
| rs9976876 | 21:36970350 | T | G | 0.46 | 0.0025 | 0.0117 | 8.33E-01 |

**Abbreviations:** OA, osteoarthritis; SNP, single nucleotide polymorphism; EAF, effect allele frequency; SE, standard error.
